# Stress perfusion Cardiac Magnetic Resonance in Patients with Antiphospholipid Syndrome

**DOI:** 10.31138/mjr.29.2.99

**Published:** 2018-06-29

**Authors:** Maria G. Tektonidou, Petros P. Sfikakis, Genovefa Kolovou, Sophie Mavrogeni

**Affiliations:** 1 First Department of Propaedeutic and Internal Medicine, Joint Rheumatology Program, Laikon Hospital, University of Athens Medical School, Athens, Greece,; 2 Onassis Cardiac Surgery Center, Athens, Greece

**Keywords:** antiphospholipid syndrome, heart disease, cardiac Magnetic Resonance, stress perfusion cardiac magnetic resonance

## Abstract

**Background:**

Antiphospholipid syndrome (APS) is characterized by the combination of recurrent arterial and venous thrombotic events and detection of persistently elevated antiphospholipid antibody titers in the serum or plasma. APS clinical manifestations also include non-thrombotic complications from various organ systems, mainly the CNS, kidneys, and heart. Cardiac manifestations of APS include valvulopathy, myocardial infarction and angina (stable, unstable, and Pritzmental angina). A previously published case series of cardiac magnetic resonance (CMR) in patients with APS has revealed a high rate of asymptomatic myocardial necrosis and scarring, but the prevalence of myocardial ischemia identified as CMR perfusion defects prior to development of necrosis is unknown.

**Aims of the study:**

To detect CMR imaging markers of myocardial ischemia in APS patients without symptoms of cardiovascular disease (CVD).

**Methods:**

We will scan fifty APS patients without symptoms of CVD stress-perfusion CMR in a 1.5 Tesla tomographer, after intravenous infusion of adenosine and gadolinium. In addition to markers of cardiac anatomy and function, we will record imaging markers of ischemia and scarring, namely perfusion defects (PDs), and late gadolinium enhancement (LGE). We will perform parametrics using dedicated software in order to derive each patient’s myocardial perfusion reserve index (MPRI). Scans will be reviewed independently by two experienced reviewers, with evaluation of inter- and intra-observer reliability. Statistical hypotheses will be examined using Student’s test and Pearson’s correlation coefficient, or non-parametric equivalents (Kruskall-Wallis and Spearman) for continuous variables, and Fisher’s exact test for binary variables. Linear or logistic regression analyses will be used to investigate APS-related determinants of subclinical myocardial ischemia.

**Anticipated benefits:**

We expect to identify CMR imaging patterns characteristic of APS, which will allow proactive therapeutic interventions for primary prevention of CVD and guide further research into the pathogenesis of APS cardiac manifestations.

## INTRODUCTION

Antiphospholipid syndrome (APS) is an autoimmune disorder characterized by venous, arterial, and/or small vessel thrombosis, pregnancy morbidity, and elevated levels of antiphospholipid antibodies (aPL), namely lupus anticoagulant (LAC), anticardiolipin antibodies (aCL), and/or anti-beta2-glycoprotein I antibodies.^1^ Any combination of thrombotic events is possible at variable intervals, and the syndrome can be either primary (PAPS) or associated with an underlying condition, most commonly systemic lupus erythematosus (SLE/APS). The 5-year mortality in APS is 5.3%, with up to 40% of deaths related to serious thromboembolic events such as stroke, pulmonary embolism and acute myocardial infarction.^2^

The most common type of cardiac involvement in APS is heart valve disease (HVD),^3,4^ characterized by thickening and/or vegetation of the cardiac valves, as initially described by Libman and Sacks in patients with SLE.^5,6^ The coexistence of antiphospholipid antibodies (aPL) with SLE is associated with a 3-fold greater risk of HVD. Valvulopathy tends to progress overtime, with a corresponding increase in the risk of stroke.

Non-valvular heart disease manifesting as myocardial infarction (MI) has been diagnosed in 5.5% of APS and was the presenting manifestation in 2.8% of APS.^2^ Additionally, coronary vasospasm, has been described as a cause of myocardial ischemia without thrombosis (also known as Pritzmental’s angina or cardiac syndrome X) in patients with APS.^7,8^ Furthermore, myocardial ischemia can be caused by thrombotic cardiac microangiopathy, with the ischemic subendocardium acting as a triggering factor for clot formation, especially in the presence of left ventricular dysfunction.^9^ Finally, endomyocardial fibrosis (EMF) has been reported as a cardiac complication of APS, possibly related to primary lesions of the coronary microcirculation.^10^ Although the pathogenesis of EMF remains uncertain, the detection of antibodies in myocardial proteins of patients with this type of pathology suggests that the autoimmune response may be implicated in its etiology.

Cardiac magnetic resonance (CMR) is a reliable, non-invasive, radiation-free technique used in the evaluation of cardiac morphology, function, perfusion, and fibrosis. Contrast-enhanced stress perfusion-fibrosis CMR using adenosine (Stress-CMR) has demonstrated very high sensitivity in the detection on subendocardial perfusion defects over conventional imaging techniques in patients with coronary disease, cardiac syndrome X, and mincroangiopathy.^11–13^ The CE-MARC study established the diagnostic accuracy of Stress-CMR for the detection of myocardial ischemia or scarring and confirmed its superiority over the single photon emission computed tomography (SPECT) and positron emission tomography (PET) scans.^14–16^

Stress-CMR is the technique of choice when quantification of scar or fibrotic tissue is needed (viability study).^17,18^ The optimal time for scar detection is between 10 and 20 minutes of contrast administration, when characterization of scar, normal myocardium and blood pooling is most accurate, based on differences in contrast enhancement. This method is termed “late gadolinium enhancement” (LGE), and it is the gold standard for the evaluation of myocardial scarring in vivo.^19^ One previous study of CMR in patients with APS has shown high rates of asymptomatic myocardial ischemia detection,^20^ introducing CMR as an emerging technique for the evaluation of indolent cardiac disease in this population.

## PATIENTS AND METHODS

Fifty patients meeting the updated Sapporo criteria for APS (2006) and followed in Rheumatology Unit of the First Propaedeutic Internal Medicine Department (Director: Professor P. Sfikakis) at Laikon Hospital, Athens, Greece, will be included in the study.^1^ Patients will be considered to have the secondary form of APS, if they have concurrent SLE, as defined by the American College of Rheumatology classification criteria.^21^ Patients with renal insufficiency, pregnancy and contraindications to CMR testing or to intravenous contrast administration, will be excluded. Informed consent will be obtained from all patients, following the protocol’s institutional review board by the local committee. Demographic, clinical and laboratory characteristics of patients will be recorded. Patients will undergo CMR on a 1.5 Tesla scanner (Signa CV/i, GE Medical Systems) using ECG-triggered steady-state, free precession breath-hold cines (echo time (TE)/repetition time (TR) 1.6/3.2 ms, flip angle 60) in long-axis planes and sequential 8 mm short-axis slices (3 mm gap) from the atrioventricular ring to the apex. Stress perfusion CMR will be performed using 140 mg/kg/min adenosine for 4 minutes (12, 20) and 0.1 mmol/kg Gd-DTPA will be given during the first-pass perfusion sequence (IR balanced Turbo Field Echo,TR 2.8 ms, TE 1.38 ms, FA 45, slice thickness 8 mm, preparation pulse delay 200 ms). A rest perfusion will be performed using the same protocol. Finally, late gadolinium enhancement (LGE) images will be acquired 10 min after intravenous gadolinium-DTPA (Schering; 0.2 mmol/kg) in identical short-axis planes using an inversion-recovery gradient echo sequence for fibrosis detection (3D-Turbo field echo sequence, TR 5.1 ms, TE 2.5 ms, FA 15, slice thickness 8 mm). Inversion times will be adjusted to null normal myocardium (typically 320–440 ms; pixel size 1.7×1.4 mm).

CMR scans will be analyzed independently by two experienced cardiologists at Onassis Cardiac Surgery Center, Athens, Greece, blinded to clinical data (S. Mavrogeni, G. Kolovou). A consensus will be used for discordant grades, and the intra and inter-observer variability will be calculated with a goal of 0.85. Ventricular volumes, anatomy, and function will be measured for both ventricles using standard techniques and analysed using specialized software. Perfusion defects (PDs) will be assessed by both visual and parametric analysis. Quantification will be performed using delineation of endo- and epicardial LV borders throughout first-pass perfusion (MEDIS system, Leiden, Netherlands). Stress and rest perfusion slopes will be derived using Fermi-fitting of signal intensity vs time and normalized to LV blood pool slope. A Myocardial Perfusion eserve Index (MPRI) will be calculated for each patient, defined as the ratio of stress to rest. Finally, LGE images will be assessed for midwall or subepicardial enhancement, compatible with myocarditis, with subendocardial or transmural enhancement in the distribution of a coronary artery, compatible with myocardial infarction, and for diffuse subendocardial fibrosis, compatible with vasculitis in this cohort.

Our main investigative hypotheses are the following:

1. There is a high prevalence of asymptomatic cardiac involvement in patients with APS, which can be readily detected using CMR. Specifically, we expect to find high rates of silent myocardial ischemia and fibrosis, manifested as subendocardial PDs, low MPRI, and abnormal LGE.
2. Distinct patterns of abnormal CMR findings will be found in PAPS and SLE/APS patients.
  Additional hypotheses include:
3. Possible correlation of PDs, MPRI, and LGE abnormalities with double and triple antiphospholipid antibody positivity, a history of arterial, venous, or dual thrombosis, the number of prior thrombotic events, a history of a history of thrombotic versus obstetric-only APS, and exposure to corticosteroids, biologic agents, or other immunosuppressants.
4. Detection of Stress-CMR abnormalities even in APS patients without traditional cardiovascular risk factor comorbidities.

## STATISTICAL ANALYSIS

Investigational hypotheses of correlation between measured variables will be carried out using Student’s test or Pearson’s correlation coefficient, or non-parametric alternatives as appropriate (Kruskall-Wallis test, Spearman’s coefficient) for continuous variables. For binary categorical variables, Fisher’s exact test will be used. Multiple linear or logistic regression analyses will be used to investigate associations of continuous or binary CMR outcome measures, respectively, with recorded demographic and clinical parameters. The pre-specified statistical significance level is set at a=0.05. STATA software version 12.0 (College Station^TM^, Texas, USA) will be used for all analyses.

## AIMS OF THE STUDY

The present study will be the first provide high-quality epidemiologic data on the prevalence of CMR-detected cardiac involvement in a representative sample of patients with APS. We will estimate the prevalence of significant lesions indicative of valvulopathy, myocardial dysfunction, microangiopathy, ischemia, or fibrosis in PAPS and SLE/APS patients, and determine the frequency of clinically silent cardiac disease in this population. We will provide detailed results on the prevalence of PDs, MPRI, and LGE abnormalities. We will perform statistical analyses to examine potential correlations of abnormal imaging findings with disease-related clinical characteristics and comorbidities. Based on the coexistence of different abnormalities in individual patients, we will describe CMR imaging patterns of cardiac involvement in APS, and detect pattern differences between PAPS and SLE/APS patients.

## ANTICIPATED BENEFITS

The present study will contribute valuable epidemiologic data on the prevalence and the patterns of asymptomatic cardiac complications in APS. This will be the first description of CMR-detected myocardial ischemia in Greek APS patients, and is expected to further our knowledge on the rarer cardiac manifestations of APS, such as microangiopathy and endomyocardial fibrosis. Finally, this will be an ideal screening for indolent cardiovascular disease in our high-risk APS population, allowing appropriate treatment and prevention of future complications.

## Abbreviations

APS: Antiphospholipid syndrome
aPL: Antiphospholipid antibodies
LAC: Lupus Anticoagulant
aCL: Anticardiolipin antibodies
PAPS: Primary APS
SLE: Systemic Lupus Erythematosus
HVD: Heart valve disease
MI: Myocardial infarction
EMF: Endomyocardial fibrosis
CMR: Cardiac magnetic resonance
Stress-CMR: Stress perfusion-fibrosis CMR
SPECT: Single photon emission computed to-mography
PET: Positron emission tomography
LGE: Late gadolinium enhancement
ECG: Electrocardiogram
TE: Echo time
TR: Repetition time
IR: Inversion recovery
FA: Fractional anisotropy
DTPA: Diethylenetriamine pentaacetate
PD: Perfusion defect
MPRI: Myocardial perfusion reserve index
LV: Left ventricle
